# Exploring the Use of a Lipopeptide in Dipalmitoylphosphatidylcholine Monolayers for Enhanced Detection of Glyphosate in Aqueous Environments

**DOI:** 10.1021/acs.langmuir.4c01089

**Published:** 2024-06-22

**Authors:** Priscila S. Ferreira, Barbara B. Gerbelli, Ana C. H. Castro-Kochi, Bruna Cortez, Fabiola L. Castro, Jorge Cantero, Federico Iribarne, Ian W. Hamley, Wendel A. Alves

**Affiliations:** † Center for Natural and Human Sciences, Federal University of ABC, Santo André 09210-580, Brazil; ‡ Theoretical Chemical Physics and Biology Group, Mathematics-DETEMA Department, Faculty of Chemistry, UdelaR, General Flores 2124, Montevideo 11800, Uruguay; § Department of Chemistry, University of Reading, Reading RG6 6AD, U.K.

## Abstract

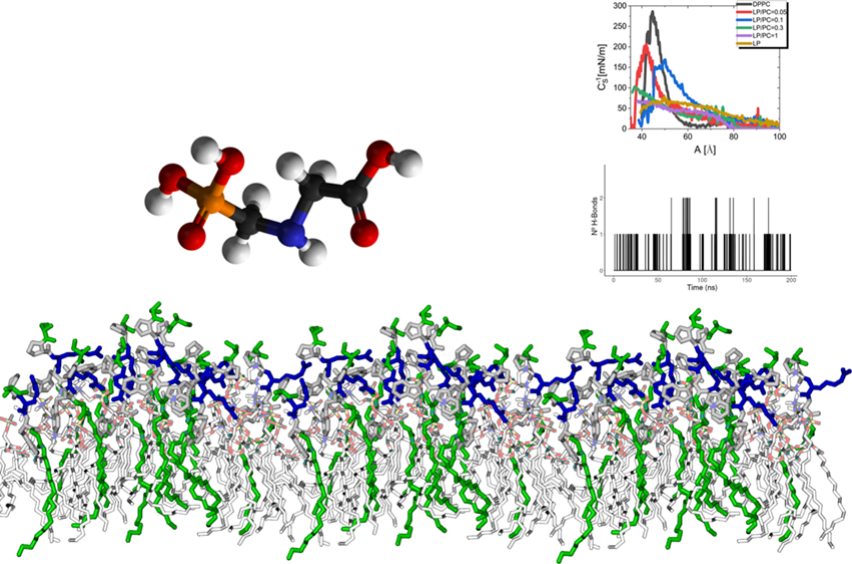

The growing reliance on pesticides for pest management in agriculture highlights the need for new analytical methods to detect these substances in food and water. Our research introduces a SPRWG-(C_18_H_37_) lipopeptide (LP) as a functional analog of acetylcholinesterase (AChE) for glyphosate detection in environmental samples using phosphatidylcholine (PC) monolayers. This LP, containing hydrophilic amino acids linked to an 18-carbon aliphatic chain, alters lipid assembly properties, leading to a more flexible system. Changes included reduced molecular area and peak pressure in Langmuir adsorption isotherms. Small angle X-ray scattering (SAXS) and atomic force microscopy (AFM) analyses provided insights into the LP’s structural organization within the membrane and its interaction with glyphosate (PNG). Structural and geometric parameters, as derived from in silico molecular dynamics simulations (MD), substantiated the impact of LP on the monolayer structure and the interaction with PNG. Notably, the presence of the LP and glyphosate increased charge transfer resistance, indicating strong adherence of the monolayer to the indium tin oxide (ITO) surface and effective pesticide interaction. A calibration curve for glyphosate concentration adjustment revealed a detection limit (LOD) of 24 nmol L^–1^, showcasing the high sensitivity of this electrochemical biosensor. This LOD is significantly lower than that of a similar colorimetric biosensor in aqueous media with a detection limit of approximately 0.3 μmol L^–1^. Such an improvement in sensitivity likely stems from adding a polar residue to the amino acid chain of the LP.

## Introduction

Pesticides are used to eliminate or regulate pests, fungi, insects, and weeds,^1^ proving beneficial for agriculture.^2^ However, it has been discovered that the use of pesticides poses a significant threat to the environment,^3^ and low but repeated exposure has been linked to various human health disorders.^4−6^ Based on their structures, pesticides can be categorized into organochlorines, organophosphates, carbamates, chlorophenols, and synthetic pyrethroids.^7^ Carbamates are a group of insecticides similar to organophosphate pesticides in structure and mechanism. The difference between carbamates and organophosphates is that carbamates bind reversibly to acetylcholinesterase, while the phosphorylation of acetylcholinesterase by organophosphates is irreversible.^8^ The most commonly used class of pesticide worldwide is organophosphate pesticides (OPs), representing 45% of the global market.^9,10^

Organophosphate pesticides act by inhibiting cholinesterases, mainly acetylcholinesterase (AchE; EC 3.1.1.7), increasing the level of acetylcholine in synapses.^11^ Pesticide applications can contaminate groundwater and rivers, causing death to people living in those areas.^12^ Their use can bolster increased resistance to these compounds in pests, necessitating higher doses and more potent products. Plants also suffer impacts from pesticide use, affecting their physical structure and metabolism.^13^ Humans, both those who handle pesticides and those who consume foods grown with these substances, are significantly harmed by its use. One of the main damages caused by pesticides to human health is the mutation of cell genes, which can later trigger cancer in various parts of the body.^14,15^ The maximum allowable limits for individual pesticides and their associated compounds are 0.1 μg/L in drinking water and 0.05 mg/kg in plant foods.^16^ The long-term presence of pesticide residues in water and agricultural products can cause severe diseases and side effects, such as Alzheimer’s, Parkinson’s, eye pain, gastrointestinal pain, seizures, respiratory failure, paralysis, and even the risk of death in humans due to their stability and increased toxicity.^17−19^

Therefore, constructing simpler, portable, inexpensive, and selective pesticide sensors is essential to finding traces in biological samples and environmental sources.^17^ Many advanced techniques have been employed for pesticide determination, including chromatographies,^20,21^ such as thin-layer chromatography (TLC),^22^ high-performance liquid chromatography (HPLC),^23^ gas chromatography (GC),^24^ and fluorometry.^25^ However, these advanced techniques are time-consuming, expensive, and challenging to be applied continuously in monitoring the use of organophosphates.^26^

In this study, we propose the development of Langmuir films composed of phosphatidylcholine and a lipopeptide (LP) to mimic the enzyme acetylcholinesterase, focusing on the interaction with the organophosphate pesticide glyphosate (N-(phosphonomethyl)glycine, PNG) and its potential application as an electrochemical biosensor. Extensive research was conducted to determine the optimal composition for the biosensor’s effectiveness. Small-angle X-ray scattering (SAXS) gave us information on how the lipopeptide influences the organization and stabilization of the vesicles in the presence of the pesticide. We investigated the adsorption isotherm of the phosphatidylcholine monolayer with varying quantities of lipopeptide and its interaction with PNG, including variations in the compressibility modulus and surface potential. Additionally, we performed atomic force microscopy (AFM), circular dichroism, and UV–visible spectroscopy experiments. The experimental procedures were complemented by molecular dynamics (MD) simulations, mainly focused on studying and quantifying the effect of LP on the structural properties of the lipid monolayer and the molecular interactions established with PNG. Through this array of powerful techniques, we developed an electrochemical biosensor based on phosphatidylcholine and lipopeptide capable of detecting PNG in aqueous solutions, achieving a detection limit of 24 nmol L^–1^. This performance is outstanding when compared to a detection limit of 0.3 μmol L^–1^ for a similar peptide used in a colorimetric biosensor, as previously described in the literature.^27^

## Materials and Methods

### General Information

**Figure 1:**
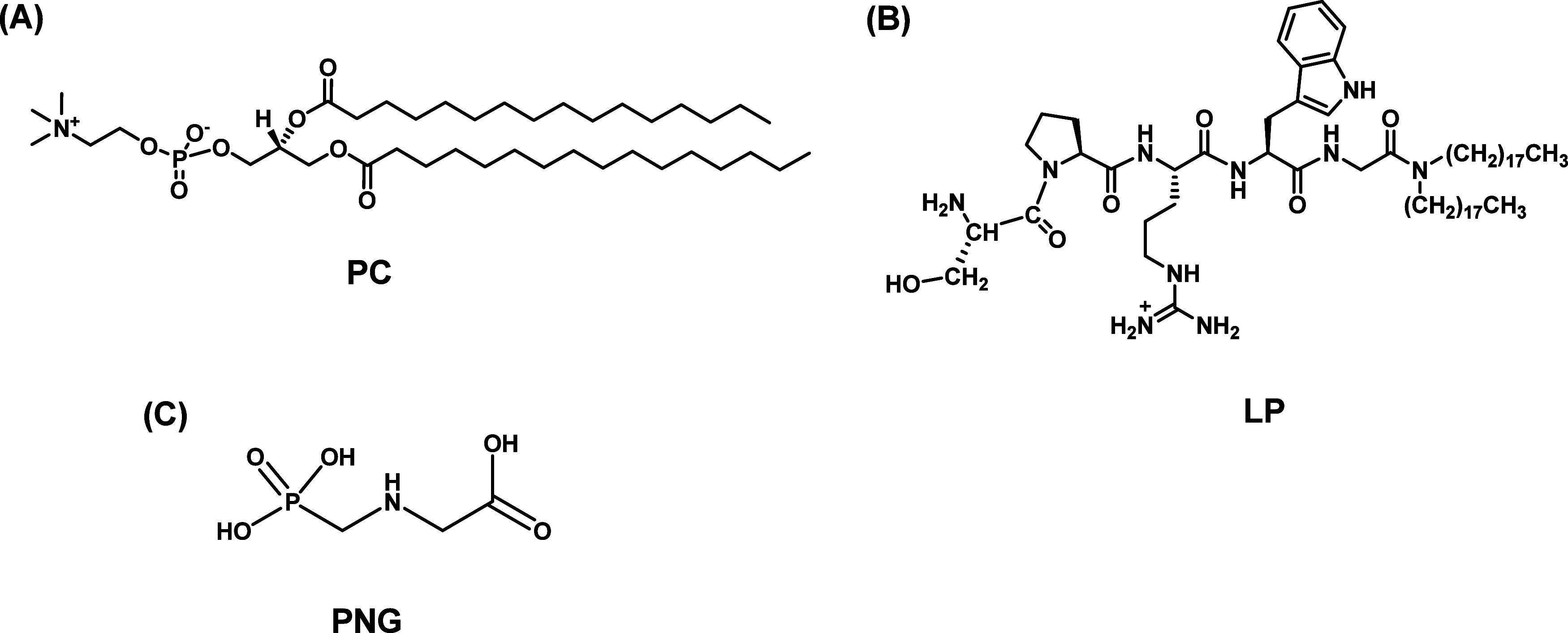
Molecular Structures: (A) 1,2-dipalmitoyl-*sn*-glycero-3-phosphatidylcholine) (**PC**), detailed structure; (B) lipopeptide SPRWG-(C_18_H_37_) (**LP**), the amino acid sequence with a hydrophobic chain; and (C) *N*-(phosphonomethyl)glycine (**PNG**) structure.

The lipid membrane was composed of a majority of 1,2-dipalmitoyl-*sn*-glycero-3-phosphatidylcholine (PC) (≥99%, Sigma-Aldrich) (Figure 1A) with a molecular weight of 734 g/mol, incorporating varying ratios of lipopeptide compound (LP) (Figure 1B). The amphiphilic molecule’s hydrophilic part consists of 5 amino acid residues: *L*-serine (S), *L*-proline (P), *L*-arginine (R), *L*-tryptophan (W), and *L*-glycine (G), covalently bonded to a hydrophobic part formed by a long aliphatic chain [SPRWG-(C_18_H_37_)]. The lipopeptides used in this study were custom-synthesized by Peptide Protein Research Ltd. (Fareham, UK). The pesticide used was an organophosphate named *N*-(phosphonomethyl)glycine (PNG) (Figure 1-C), solubilized in Milli-Q water (Type 2) at varying concentrations. PC was prepared in a 1 mg mL^–1^ stock solution solubilized in chloroform, while LP was solubilized in methanol and chloroform 50:50, and a 1 mg mL^–1^ stock solution was prepared. Different molar ratios of LP/PC ranging from 0.05 to 1 were used. The PNG concentration was varied for each monolayer from 1 to 15 μmol L^–1^. The Langmuir trough, Langmuir–Blodgett (LB), and AFM techniques were employed for monolayer characterization.

### Small-Angle X-ray Scattering

Small-angle X-ray scattering (SAXS) experiments of lipid vesicles, both in the absence and presence of varying lipopeptide concentrations, were conducted at the BioSAXS beamline B21, Diamond Light Source, UK. Lipid solutions were loaded into a 96-well plate of the EMBL BioSAXS robot. These solutions were then automatically injected through an automated sample changer into a 1.8 mm internal diameter quartz capillary, where they were exposed to the X-ray beam. Approximately, 20 frames were acquired for each sample with a continuous sample flow through the capillary. The B21 beamline operated with a sample–detector distance set at 3.9 m and a wavelength (λ) of 1.00 Å. Image collection was facilitated using a Pilatus 2 M detector. Subsequent data processing tasks, including background subtraction and radial averaging, were performed using the dedicated ScÅtter software for the beamline.^28^

### Circular dichroism and UV–Vis Spectroscopy Experiments

Circular dichroism (CD) spectra were recorded as described previously.^27^ CD and UV–visible were collected simultaneously to guarantee a direct comparison of all experimental results.

### Langmuir Trough Experiments

To explore the interaction between phosphatidylcholine (PC) and various lipopeptide (LP) ratios within the monolayers and to examine the adsorption isotherm, experiments were conducted in a Langmuir trough (KSV Instruments) at a constant temperature of 21 °C. The trough’s subphase was consistently filled with 190 mL of Milli-Q water. Different [LP/PC] ratios, ranging from 0.01 to 1, were prepared and spread on the subphase surface. After application, a 15 min waiting period was observed to ensure the complete evaporation of the organic solvents. Several compression and decompression cycles were performed for each experiment at a 15 mm/min speed. PNG was introduced into the aqueous subphase 30 min later to allow the monolayer to stabilize at a surface pressure of 30 mN/m.

We maintained constant pressure (30 mN/m) for 30 min to enhance the interaction between the pesticide and the monolayer. Subsequently, another set of six compression and decompression cycles was carried out. Supplementary surface potential measurements were conducted using the KSV NIMA surface potential setup.

### Atomic Force Microscopy

AFM measurements were conducted on monolayers after transferring to mica substrates using the LB technique. This method ensures that the nonpolar sections of the lipid films are oriented upward, which is crucial for accurate structural analysis. For these experiments, we employed a Bruker MultiMode VIII instrument, part of the NanoScope V series, located at the National Nanotechnology Laboratory in Campinas (LNNano), Brazil. The measurements were carried out in the air to stabilize the monolayer and prevent any disturbances that could arise from fluid dynamics. This setup helps achieve high-resolution topographical analysis critical for assessing the structural effects of LP and PNG incorporation within the monolayer.

AFM analyses were performed in tapping mode using a silicon tip with a force constant of 2.8 N/m and a resonance frequency of approximately 75 kHz. Scans covered areas between 0.5 and 2.0 μm^2^ at a resolution of 512 × 512 pixels. Topographical and phase data were processed and interpreted using the Gwyddion software package,^29^ allowing detailed visualization and quantification of the monolayer’s structural properties. This method provides essential insights into the arrangement and stability of lipid films, particularly in the context of our biosensor’s functionality.

### Electrochemical Characterization

Electrochemical investigations used indium tin oxide (ITO) as the conductive substrate. We employed the Langmuir–Schaefer (LS) method to transfer the monolayer onto the substrate. This technique focuses on the horizontal collection of the film, emphasizing the positioning of the film’s polar part upward (Figure S1).

For modifying the ITO electrode with the monolayer, 6 μL of PNG with varying concentrations of 1, 5, 8, 10, and 15 mmol/L, both in the presence and absence of the pesticide, was added and a 20 min waiting period ensured the complete drying of the PNG solution, followed by a careful wash to remove excess pesticide on the electrode’s surface.

Electrochemical readings were performed using the Metrohm Autolab PGSTAT 302N system, which was equipped with FRA2 and operated using NOVA 2.1.3 software. A conventional three-electrode system was installed: the ITO electrode modified with the lipidic monolayer functioned as the working electrode, a platinum wire served as the counter electrode, and a silver/silver chloride (Ag/AgCl (3 mol/L KCl)) electrode acted as the reference against which all potentials were measured. The assays were conducted in a 0.1 mol L^–1^ KCl solution (pH 7.3) containing 5 mmol L^–1^ of the redox probe K_4_Fe(CN)_6_/K_3_Fe(CN)_6_. Electrochemical impedance spectroscopy measurements occurred in the electrolytic solution at the half-wave potential *E*_1/2_ derived from prior cyclic voltammetry (around 240 mV), covering a frequency range from 0.1 Hz to 30 kHz.

### Molecular Dynamics Simulations

The lipid monolayer model, composed of 40 PC units, was initially built on a surface of 50.20 × 50.20 Å, defining a total lipid surface area of 2520 Å^2^. The monolayer surface was prepared using the charmm-gui interface.^30^ For the systems where LP was present, some PC units in the monolayer were replaced by the corresponding SPRWG-(C_18_H_37_) or PRWG-(C_18_H_37_) compounds, distributing the molecules over the membrane until reaching a 30% molar concentration. The starting orientation of LP was assigned by introducing the aliphatic tail in parallel to the lipid tails of PC molecules, with the peptide moiety protruding from the monolayer surface. After that, monolayers were solvated in water, and PNG was located at the center of the simulation box, 12 Å away from the corresponding lipid surfaces.

LP parameters (peptide and lipid regions) were selected using the Charmm36 force field.^31^ The PNG ligand was parametrized with CHARMM general force field (CGenFF)^32^ and subsequently optimized with ffparam,^33^ following the CHARMM parametrization criteria. MD simulations were undertaken with the NAMD 2.14 package.^34^ Trajectories were generated under periodic boundary conditions and an isothermal–isobaric ensemble (NPT) at a constant temperature of 310 K. The integration time step was 2 fs, the nonbonded cutoff radius was set to 12 Å, and the long-range electrostatic interactions were described utilizing the PME algorithm. Simulation cells were considered flexible, keeping the unit cell ratio constant in the *x*–*y* plane. To ensure the correct equilibration of the systems, 6 cycles of minimization and relaxation were carried out, in which the system constraints were gradually reduced until vanishment. The ensuing production stage proceeded without restrictions, for a total period of 200 ns and a systematic sampling every 10 ps. The analysis phase of simulations was assisted with VMD software,^35^ in particular, the molecular visualization and processing of trajectories.

## Results and Discussion

**Figure 2:**
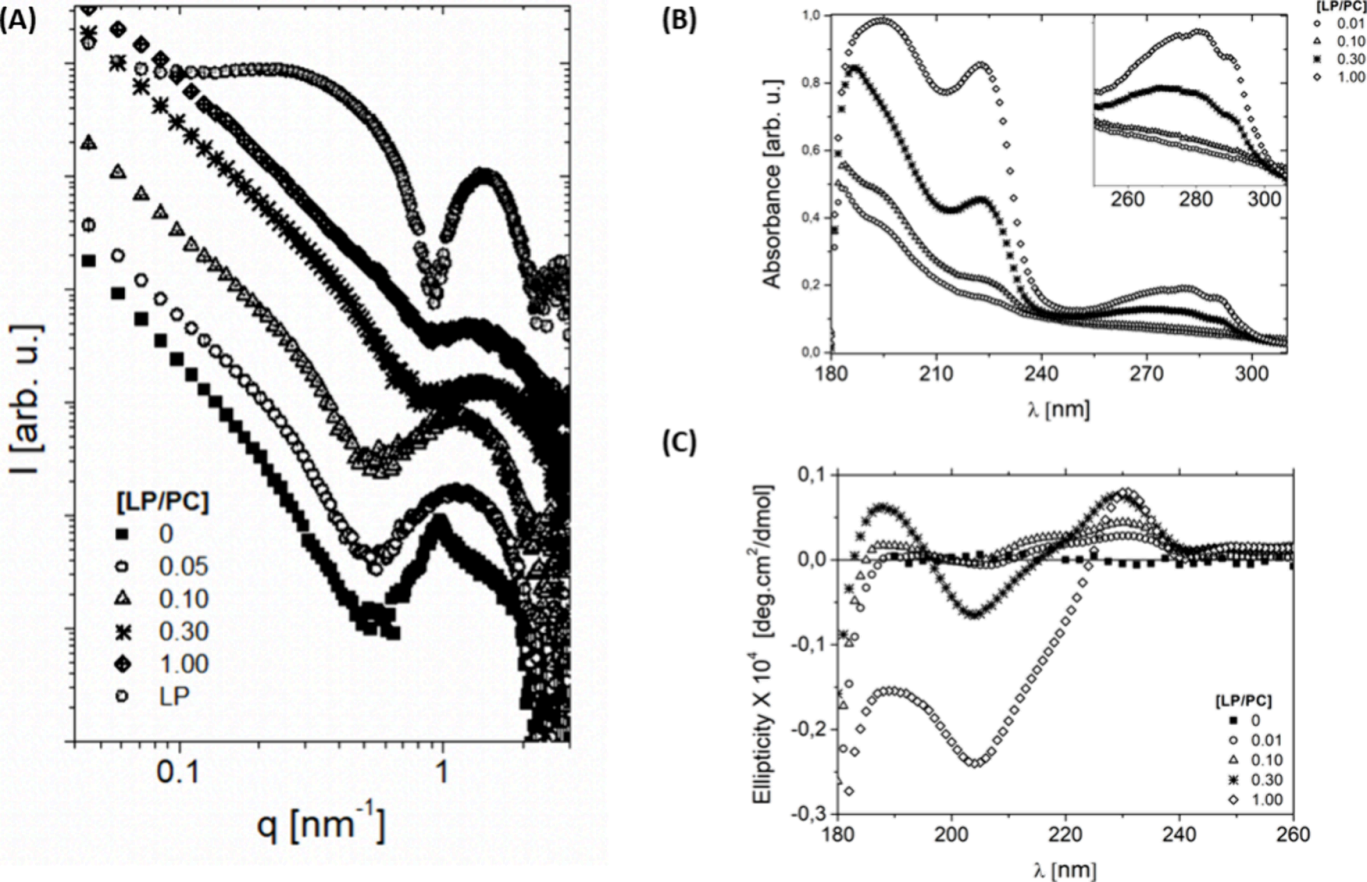
(A) SAXS data, (B) UV–visible spectra, and (C) circular dichroism data for various LP/PC molar ratios. The square symbol represents a molar ratio of 0 (PC only), the circle symbol denotes a ratio of 0.05, the upward triangle signifies a ratio of 0.10, the star corresponds to a ratio of 0.30, the diamond indicates a ratio of 1.00, and the sphere symbolizes pure LP.

Vesicles were prepared with various concentrations of lipopeptides to explore their effect on phosphatidylcholine (PC) layers. SAXS experiments were performed to investigate the structural dynamics of this lipid complex system (Figure 2A). This technique facilitates the assessment of the lipid bilayer morphology in the presence of lipopeptides and any resultant structural modifications. In Figure 2A, the scattering intensity for the system composed exclusively of PC (black square) exhibits a decrease in the low *q* range with a slope of −2, and characteristic of planar systems such as vesicles.^36^ Additionally, a Bragg peak observed around 0.95 nm^–1^ corresponds to a lamellar periodicity of 6.6 nm, further detailing the organized structure of the lipid assembly.

In contrast, the system containing only the lipopeptide (LP) is consistent with previously reported findings, indicating the formation of spherical micelles with a core–shell structure.^37^ Adding lipopeptides to the vesicles results in the disappearance of the Bragg peak, suggesting that the lipopeptides facilitate a transition from multilamellar to unilamellar vesicles.^38^ Introducing single-chain molecules into the lipid systems creates local topological defects, which enhance flexibility and favor the development of unilamellar vesicles.^39^ As the concentration of lipopeptide in the membrane increases, a significant change in the form factor scattering is observed, shifting toward the minimum value seen in systems composed exclusively of LP. Notably, even at the highest LP/PC ratio, the characteristic −2 exponential decay of intensity remains, suggesting that its planar conformation is preserved despite considerable alterations in the bilayer internal structure.

We conducted UV–visible spectroscopy and circular dichroism experiments for the same LP/PC molar ratios (Figure 2B). The UV–visible spectra reveal that as the amount of LP in the lipid vesicles increases, two absorption bands emerge between 180 and 240 nm. Literature suggests that the presence of these bands is associated with the formation of π–σ* or π–π* type bonds.^40^ Additionally, for LP/PC ratios of 0.30 and 1.00, the spectral signature related to tryptophan is observable in the region from 260 to 300 nm.^41^ Circular dichroism data further confirm the formation of structures indicative of π–π* interactions. With an increase in the LP/PC molar ratio, the emergence of peaks at 190 and 205 nm becomes very pronounced, characteristic of the formation of β-sheet type structures undergoing a blue shift (Figure 2C).

**Figure 3:**
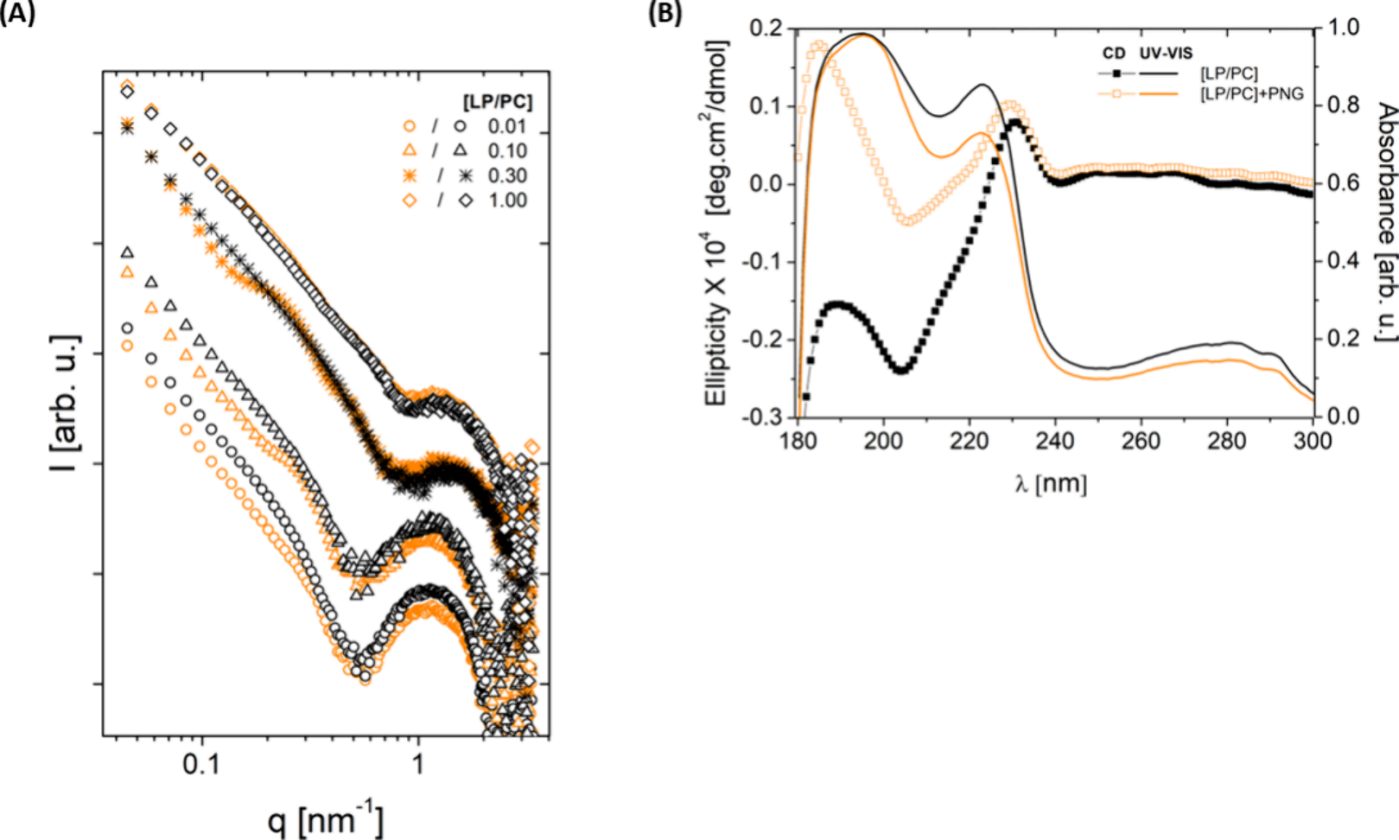
(A) SAXS data, (B) circular dichroism spectra, and UV–visible spectra for various LP/PC molar ratios in the presence of PNG. Black symbols represent the system without the pesticide, and orange symbols indicate the presence of PNG in the aqueous solution.

We replicated the previous experiments in the presence of *N*-(phosphonomethyl) glycine (PNG), adding 15 μmol of PNG to the aqueous solution for each LP/PC molar ratio. As previously reported, we allowed 10 min for equilibration before collecting all data.^27^ The vesicles decrease in size for compositions with an LP/PC molar ratio less than 0.10, whereas those with an LP/PC ratio greater than 0.30 maintain their size upon adding the pesticide (Figure 3A). A significant change in conformation is observed with the addition of PNG to the solution, as demonstrated in Figure 3B, where the observed CD signal is characteristic of a structure with a helical conformation, presenting a positive band around 195 nm and two negative bands around 209 and 223 nm. This may be related to the increase in available surface charge on the membrane due to the addition of PNG, which was subsequently confirmed with the measurements of surface potential variation for LP/PC ratios of 0.01 and 1 at various PNG concentrations (see Table 1), to be discussed later in the text.

**Table 1:** Surface Potential Variation for LP/PC Ratios of 0.10, 0.30, and 1.00 at Various PNG Concentrations^a^

	[LP/PC] = 0.10	[LP/PC] = 0.30	[LP/PC] = 1.00
[PNG] (μmol L^–1^)	Δ*SP* (mV)	Δ*SP* (mV)	Δ*SP* (mV)
1	0.01		0.09
5	0.02	0.11	0.18
10	0.05	0.12	0.80
15	0.08	0.20	0.50

a Δ*SP* indicates the difference in surface potential values between conditions with and without the presence of the pesticide, demonstrating the increasing interaction strength as the LP concentration in the monolayer increases.

**Figure 4:**
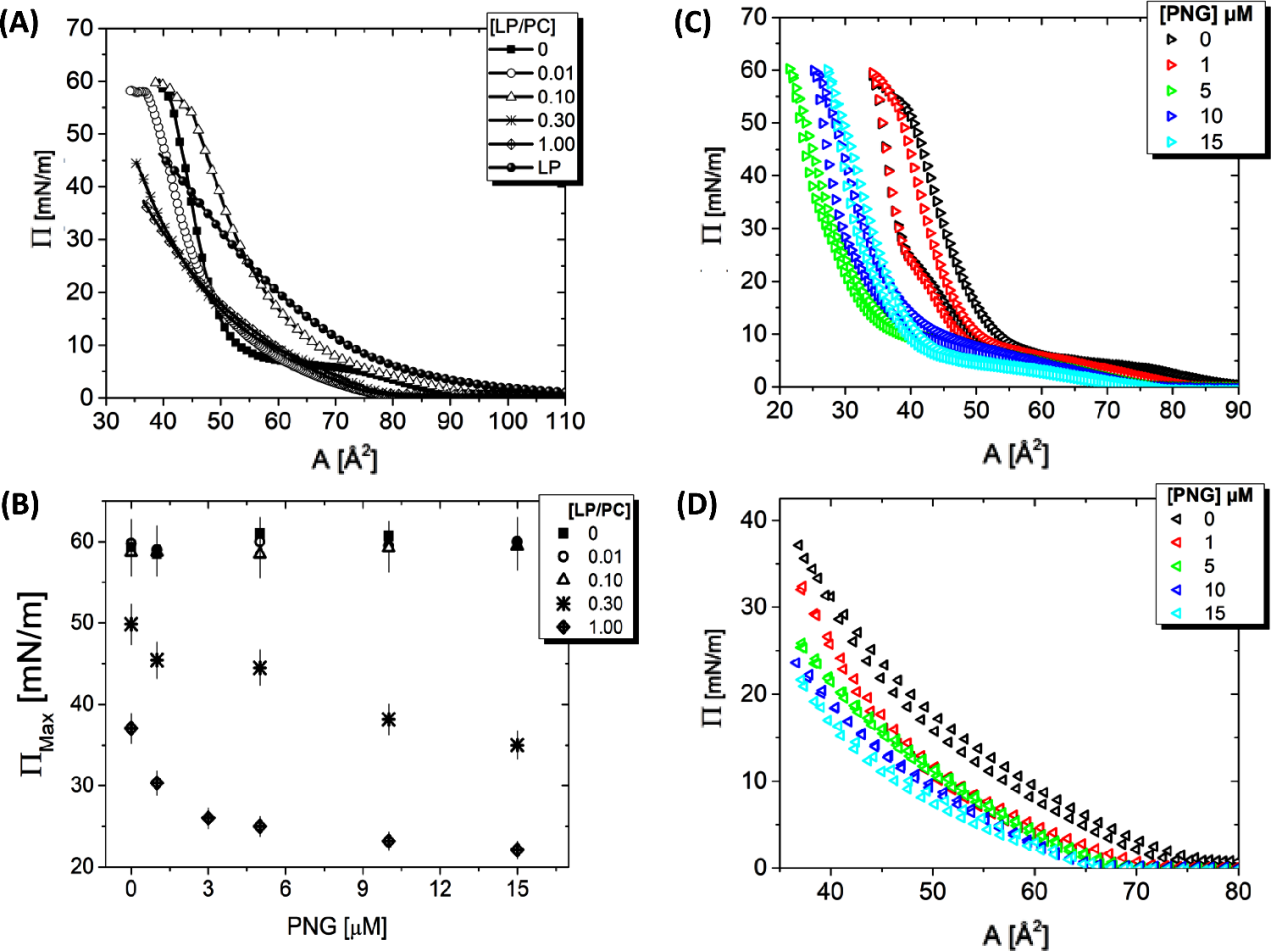
Illustration of the characteristics of monolayers under various conditions. (A) Surface pressure isotherms for different LP/PC ranging from 0.05 to 1.00. (B) Maximum surface pressure as a function of PNG concentration for each LP proportion in the PC monolayer. (C) Adsorption isotherms at an LP/PC molar ratio of 0.01, with PNG concentrations varying from 1 to 15 μmol L^–1^. (D) Adsorption isotherm at an LP/PC molar ratio of 1.00, illustrating the effect of different PNG concentrations on the monolayer.

By examining Langmuir monolayers, we assessed the impact of varying lipopeptide (LP) concentrations on phosphatidylcholine (PC) within the lipid membrane. The molecular area-pressure isotherm for the pure PC monolayer displays characteristic behavior, as documented in the literature.^42−45^ With increased LP concentration within the lipid monolayer, we note a decrease in the average molecular area and a reduction in maximum surface pressure (Figure 4A). This observation aligns with the properties of LP, which, as a single-chain molecule,^46^ occupies a smaller molecular area than pure PC.^47^

In Figure S2, the time evolution of the monolayer thickness is illustrated for both a pure PC membrane (Figure S2A) and a system with an LP/PC molar ratio of 0.30 (Figure S2B). The data shows that the monolayer is slightly thicker in the pure PC configuration, with average thickness values of 21.1 Å compared to 20.1 Å when LP is incorporated. Consequently, the total monolayer area decreases with adding LP, corroborating the experimental findings. Additionally, the pure PC monolayer exhibits smaller fluctuations in thickness over time (standard deviations of 0.4 Å) compared to the system containing LP (0.7 Å), suggesting a more compact structure in the absence of LP. Molecular models of the pure PC and [LP/PC] = 0.30 lipid monolayers are shown in Figure S3A,B, respectively.

Figure 4B shows the maximum surface pressure (Π_Max_) as a function of PNG concentration for each ratio of LP present in the PC monolayer. At a LP/PC ratio of 0.01, a slight distinction between pure PC and LP/PC behavior becomes noticeable even at low molar concentrations of PNG (Figure 4C). However, for the [LP/PC] = 1.00 monolayer, an increase in PNG concentration decreases the maximum pressure, stabilizing at approximately 23 mN/m (Figure 4D). As the pesticide concentration increases, this difference becomes more pronounced.

For the composition [LP/PC] = 1.00, the difference in Π_Max_ is more accentuated, facilitating the determination of the necessary percentage of LP in the monolayer to detect a specific minimum concentration of PNG in an aqueous solution (Figure 4B). These findings indicate that it is possible to ascertain the optimal percentage of LP in the monolayer to detect a specific minimum concentration of PNG in an aqueous solution.

The reduction in maximum surface pressure values at concentrations of 0.30 and 1.00 is attributed to enhanced interaction between the monolayer and PNG molecules, suggesting a more significant bonding affinity. This increased interaction correlates with the alterations observed in the monolayer compressibility modulus (*C*_S_^–1^), which can be determined using eq 1:
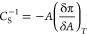
1

This thermodynamic parameter provides insights into the flexibility of lipid films and the configuration of the carbon chain within the monolayer. Phosphatidylcholine (PC) forms a more rigid structure than other compositions, as depicted in Figure S4. We identify the expanded liquid (LE) phase in the range of 12.5 < *C*_S_^–1^ < 50 mN/m and the condensed liquid (LC) phase within 100 < *C*_S_^–1^ < 250 mN/m, which are characteristic of a pure PC monolayer.^44^ Incorporating lipopeptides (LPs) into the monolayer results in a notable reduction in *C*_S_^–1^ values, reaching approximately 60 mN/m, indicative of the LE phase (Figure S4).^48,49^ This alteration in *C*_*S*_^–1^ suggests the transition to a less rigid monolayer, with increased flexibility attributed to the single-chain LP, which introduces defects that render the monolayer more fluid.^50,51^ In contrast, the pure LP monolayer exhibits a *C*_S_^–1^ behavior that nearly forms a plateau, indicating a distinct mechanical property profile.

The so-called area per lipid (APL) is another interesting parameter that has been computed during the analysis stage of the theoretical simulations to evaluate the rigidity of the monolayer systems. The results presented in Figure S5 reveal a sharp increase in APL at the [LP/PC] = 0.30 ratio (Figure S5B). Effectively, the APL average value climbs from 47.5 to 67.2 A°^2^ upon introduction of LP. This translates into a more fluid and flexible membrane, according to the compressibility modulus results. Individual lipid molecules can move around more freely with extra space available. The APL results are in line with those found for monolayer thickness. Naturally, as the lipids spread horizontally to occupy more area, the width of the membrane is reduced.

Figure 4D illustrates an adsorption isotherm for the monolayer at a 1.00 LP/PC molar ratio, with the PNG concentration varying from 1 to 15 μmol L^–1^. As the concentration of PNG in the medium increases, the molecular area decreases. This phenomenon is likely due to the pesticide binding to the hydrophilic region of the monolayer, resulting in electrostatic interactions that reduce the molecular area occupied per molecule, thereby fostering a more compact monolayer structure. For the [LP/PC] = 1.00 monolayer, increasing PNG concentration decreases maximum pressure, stabilizing at approximately 23 mN/m (Figure 4D). The introduction of pesticides is known to destabilize the monolayer, a behavior consistent with observations made in the literature in the presence of antibacterial molecules.^52^ To elucidate the structural changes within the monolayer, Kralchevsky and colleagues introduced a new model for charged surfaces.^53^ In Figure S6, the data from the nonhorizontal portion of the isotherms shown in Figure 4C are replotted as Π vs A^–3/2^. Here, a decrease in the slope value is observed as the pesticide concentration increases. According to the authors, this decrease in Π following an increase in pesticide concentration in the aqueous solution could be attributed to the disaggregation of molecules, suggesting a possible explanation for the observed reduction in monolayer stability (Figure S6).

Surface potential (Δ*SP*) measurements represent another critical technique for characterizing Langmuir films.^54^ The values obtained for the pure PC monolayer align with those reported in the literature.^55^Figure S7 provides insights into the surface potential of the PC monolayer across different LP/PC ratios at a PNG concentration of 15 μmol L^–1^. A decrease in SP variation is noted as the pesticide is introduced to the aqueous subphase. Given that PC inherently carries a negative electrostatic charge, the combination of LP and PNG with PC results in a notable shift in the surface charge, which changes from negative to positive.

Table 1 presents the Δ*SP* values across three distinct LP/PC ratios: 0.10, 0.30, and 1.00, demonstrating the change in surface potential in response to the presence of the pesticide. The data reveal a minimal change at the [LP/PC] = 0.10 ratio, suggesting a weaker interaction with the pesticide. However, as the LP concentration increases to 0.30, there is a noticeable enhancement in Δ*SP* values, which becomes even more pronounced at the [LP/PC] = 1.00 ratio, where the Δ*SP* value shows an approximately 10-fold increase compared to the lowest concentration. This apparent trend underscores that higher concentrations of LP molecules in the monolayer significantly boost the interaction strength with the pesticide, highlighting the efficacy of LP in facilitating stronger pesticide interactions.

To acquire more direct evidence of the role of LP as a mediator of the molecular interaction of PNG with the lipid membrane, the radial distribution function (*g*(*r*)) was measured throughout the MD simulations (Figure S8). This function is a valuable tool for understanding the way species are arranged and located from one another in a given system. Its value gives the probability of finding two particles at a certain distance. The *g*(*r*) probability in Figure S8 shows that the most frequent distance between the PNG center of mass and the LP serine residue is around 5.5 A°, with a probability over 1.5. On the other hand, for the pure PC membrane (Figure S8A), the density peak (barely more significant than 1) corresponds to a distance of nearly 7.5 A°. These results indicate that PNG tends to lie much closer (and more frequently) to the monolayer when LP is incorporated, endorsing the notion of a more robust interaction of PNG with the membrane (Figure S8B).

To delve deeper into the interaction between PNG and the monolayer, we calculated the nonbonded (electrostatic and van der Waals) energies for the systems, again with an LP/PC molar ratio of 0.0 or 0.30. Results are summarized in Table 2 and demonstrate that both components (i.e., van der Waals and electrostatic) of the nonbonded interaction are magnified for the system with [LP/PC] = 0.30. Overall, the total nonbonded energy of PNG in the presence of LP amounts to −18.26 kcal/mol (sum of columns 3 and 4 of the corresponding system in Table 2) as opposed to −7.95 kcal/mol for the pure PC system. The 10 kcal/mol gap favoring the former underpins the radial distribution function results and previously discussed surface potential measurements.

**Table 2:** Average Electrostatic (Ele), van der Waals (Vdw), and Total Non-bonded Interaction Energies (in kcal/mol Plus Standard Deviations) between PNG and Monolayer for All Simulated Systems

[LP/PC]	Type	PC	LP
0.0	Vdw	–2.46 ± 3.3	
	Ele	–5.49 ± 9.7	
	Total	–7.95 ± 12	
0.30	Vdw	–1.83 ± 2.7	–5.33 ± 6.4
	Ele	–4.42 ± 8.9	–6.68 ± 13
	Total	–6.25 ± 11	–12.01 ± 16

**Figure 5:**
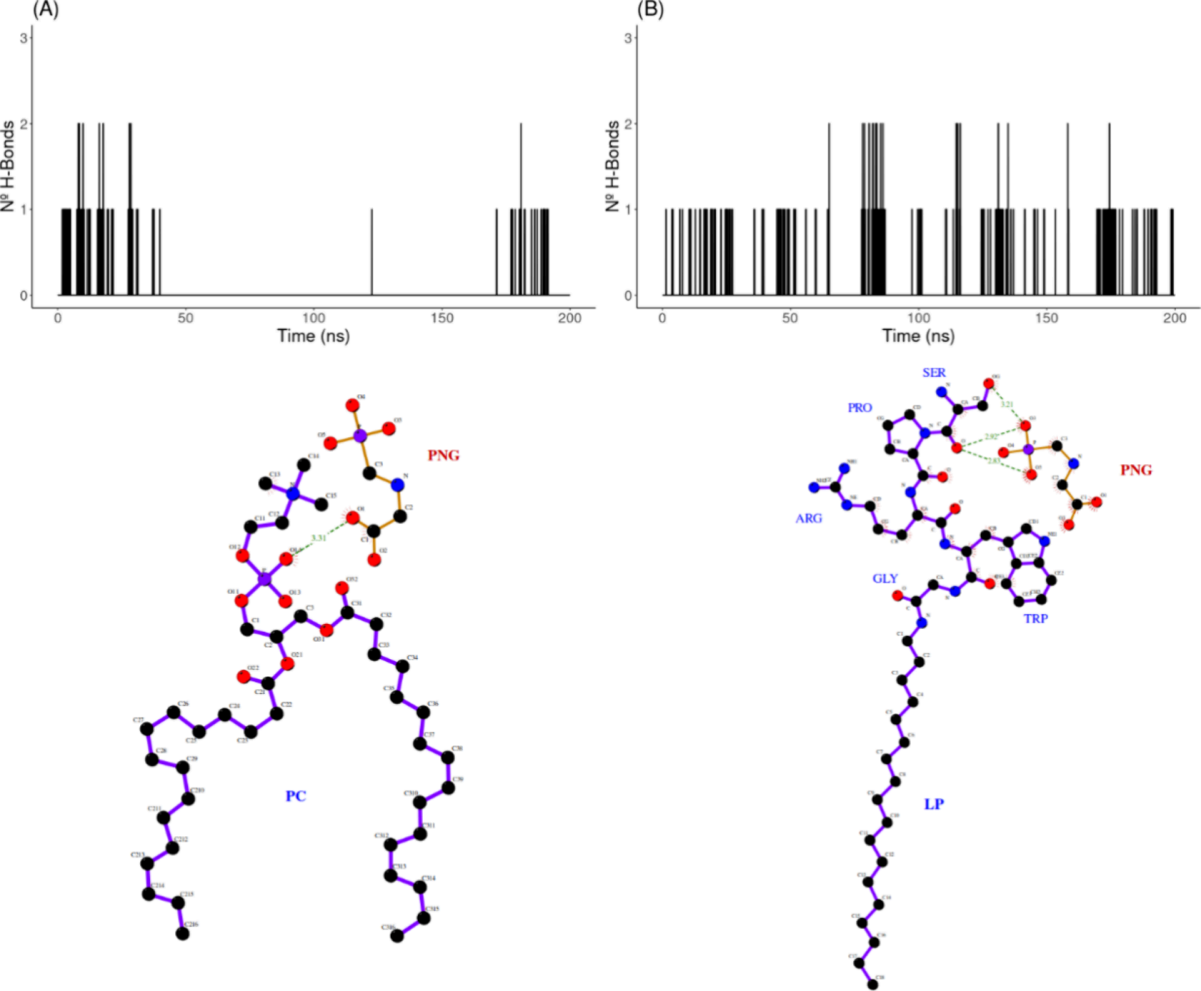
Time evolution (up) and 2D molecular rendering (down) of the number of H-bonds established between PNG and the lipid monolayer along the MD trajectories of simulated systems. (A) pure PC monolayer and (B) lipid monolayer at an [LP/PC] = 0.30 ratio. In 2D rendering, distances between the potential H-bond acceptor and donor atoms in PNG and the lipid monolayer are indicated for a representative time step in the simulation.

Owing to the polar nature of PNG, the formation of hydrogen bonds (H-bonds) should make an essential contribution to the interaction with the monolayer. Bearing this in mind, the occurrence of H-bonds was analyzed over all the MD trajectories, and the results are graphed in Figure 5. H-bond patterns show that in the presence of LP ([LP/PC] = 0.30), more bonds are formed and much more frequently in time in comparison to the pure PC monolayer. While the establishment of H-bonds is distributed along the simulation trajectory with the [LP/PC] = 0.30 monolayer, there is virtually no H-bond formation for nearly 150 ns (out of 200) for that of the pure PC monolayer (compare plots (A) and (B) in Figure 5). This outcome came as no surprise since one would expect that H-bonds involving PNG would have a higher probability of taking place with the polypeptide moieties of the LP molecules (which are located on the surface of the monolayer) rather than with the PC molecules themselves, which exhibit a less polar nature because of the aliphatic chains. Such a scenario is showcased in the 2D molecular rendering of Figure 5. The H-bond results are consistent with the interaction energy and radial distribution function results presented above.

**Figure 6:**
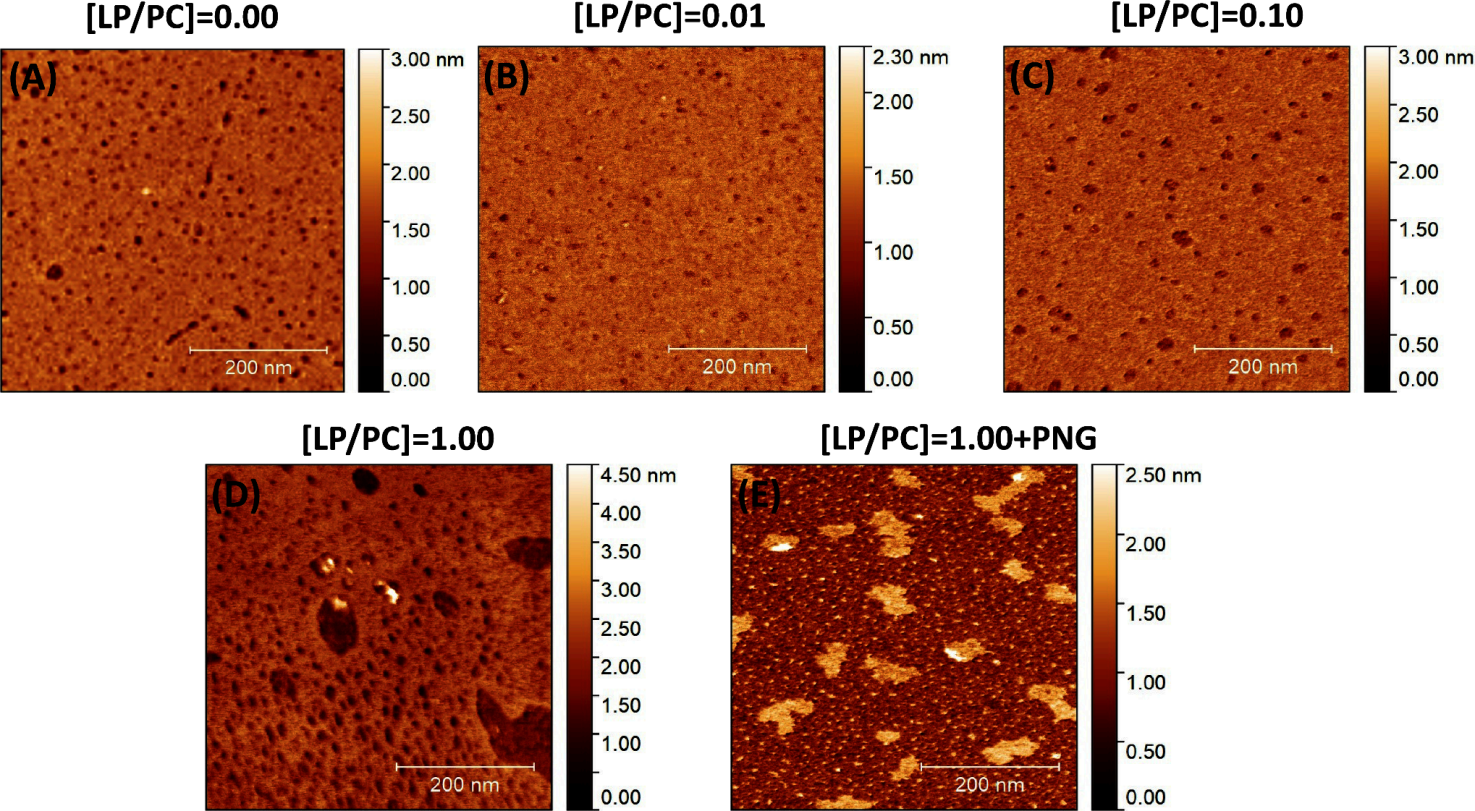
AFM images of LB films under different conditions: (A) [LP/PC] = 0.00, representing the pure PC monolayer as a control; (B) [LP/PC] = 0.01; (C) [LP/PC] = 0.10; and (D) [LP/PC] = 1.00 without PNG; (E) [LP/PC] = 1.00 with the addition of PNG in the aqueous solution.

AFM was employed to investigate the characteristics of lipidic monolayers (Figure 6). Prior to the detailed observations, it is important to note that the AFM measurements were performed under specific conditions to preserve the structural integrity of the monolayers.^56−58^ Unlike many setups that utilize AFM in fluid environments, our experiments were conducted in air to avoid the fluctuations and instabilities associated with fluid dynamics. This choice was crucial for observing the monolayer topography with high resolution and stability, particularly after incorporating the LP and the pesticide PNG. The samples were prepared using the LB technique to transfer the monolayers onto mica substrates, ensuring proper and consistent orientation of the layers. The inclusion of LP and PNG was performed during the formation of the Langmuir film on the water surface, allowing for a uniform distribution within the lipid matrix before transfer to the mica.

As shown in Figure 6A, the image obtained for the pure PC monolayer is consistent with the literature and serves as a control to demonstrate the baseline physical structure of the lipid film without LP.^59−61^ For the composition [LP/PC] = 0.01, Figure 6B displays a uniform LB film with minor localized disruptions scattered across the image. As the proportion of LP increases to [LP/PC] = 0.10, observed in Figure 6C, there is a noticeable increase in the density and size of these disruptions. This trend continues and becomes even more pronounced at the highest tested concentration, [LP/PC] = 1.00, as depicted in Figure 6D, where the disruptions are significantly larger than those in lower LP concentrations. Additionally, Figure 6E illustrates the LB film for [LP/PC] = 1.00 following the addition of PNG in the aqueous solution, highlighting further changes in the monolayer structure influenced by the interaction with the pesticide.

The introduction of lipopeptides into the PC membrane led to significant alterations in the structure of the lipid film, as evidenced by the increased disruptions visible in the AFM images from Figure 6B,D. These structural changes were exacerbated by adding the pesticide to the [LP/PC] = 1.00 monolayer, resulting in complete membrane destabilization, as illustrated in Figure 6E. This profound effect is likely due to the enhanced interaction between the lipopeptide-enriched monolayer and the pesticide, reinforcing our hypothesis about the sensitivity of the LP/PC matrix to environmental changes. Supporting this visual evidence, the molecular area data also revealed decreased electrostatic interactions and reduced surface pressure with increasing LP concentration. Additionally, the compressibility modulus of the monolayer was altered, indicating that the structural integrity and mechanical properties of the monolayer are significantly influenced by both the molar ratio of LP to PC and the presence of the pesticide.

The prior characterization of monolayers was crucial in selecting the optimal membrane composition for constructing the biosensor. After comparing all compositions and their structural and thermodynamic parameters, the [LP/PC] = 0.30 ratio was chosen. This decision was based on the observed differences in surface pressure, which suggested a favorable interaction between the monolayer and the pesticide. Moreover, this specific composition maintained satisfactory surface pressure and rigidity for LS assays. As detailed in the Materials and Methods section, indium tin oxide (ITO) was employed as the conductive substrate. By comparing AFM images of the ITO surface without and with the monolayer, it was determined that approximately 56.5% of the area was covered by the monolayer (Figure S9).

**Figure 7:**
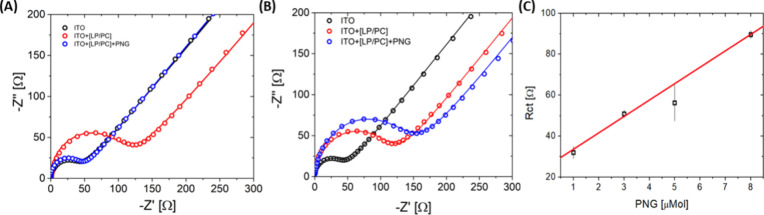
Electrochemical analysis illustrating the modification of ITO with an LP/PC monolayer at molar ratios of 0.05 (A) and (B) [LP/PC] = 0.30 upon the addition of PNG. (A, B) Nyquist plots show the response of unmodified ITO (black line), ITO modified with the monolayer (red line), and ITO further modified with the monolayer in the presence of PNG (blue line). (C) Calibration curve for detecting the pesticide PNG using an LP/PC ratio of 0.30.

Building on previous observations of the complex formed by PC and LB films and complemented by AFM analyses, the Nyquist plots (Figure 7A,B) offer further insights into the interaction dynamics within the monolayer. An observed increase in resistance to charge transfer occurs after modifying the ITO surface with the presence of the monolayer and the subsequent addition of the pesticide. This response hints that the monolayer effectively adheres to the ITO surface, and the pesticide engages with the monolayer, corroborating the destabilization and structural changes previously identified in the AFM images.

To evaluate the performance of an LP/PC-based electrochemical biosensor for detecting PNG in water, monolayer deposition was conducted using the LS technique on ITO-glass surfaces. Subsequent electrochemical tests were carried out to assess the sensor functionality, including cyclic voltammetry (CV), and electrochemical impedance spectroscopy (EIS).

For the analysis of semireversible and diffusion-controlled systems, the Randles-Sevcik equations (Equations S1 and S2) were applied to calculate the electroactive surface area of the electrodes modified with the monolayer from the data in Figure S10, with the results presented in Table 3. This approach aids in understanding the efficiency of the electrode surface modifications for improved detection capabilities.

**Table 3:** Electroactive Area of ITO Electrodes Was Modified with a Monolayer at an LP/PC Molar Ratio of 0.30, Showing Variations in Response to Different PNG Concentrations

[PNG] (μmol L^–1^)	Electroactive Area (10^–2^ cm^2^)
0 (in ITO)	2.00
1	0.29
3	0.24
5	0.32
8	0.31
10	0.32

The findings indicate that the concentration of PNG at an LP/PC molar ratio of 0.30 does not significantly affect the biosensor’s electroactive area calculation, suggesting that the selected molar ratio is optimal for the system and boosts its sensitivity.

Figure 7A shows that with a platform at a 0.05 LP/PC molar ratio, there was no discernible change in charge transfer resistance after adding PNG. Conversely, for the indium oxide doped with tin (ITO) surface modified with a monolayer at a 0.30 LP/PC molar ratio, an increase in resistance to charge transfer was noted upon introducing PNG (Figure 7B). This result indicates the biosensor’s ability to detect the presence of PNG effectively.

Furthermore, a calibration curve was established by adjusting the PNG concentration (Figure 7C), yielding a detection limit of 24 nmol L^–1^. This result was attained with an [LP/PC] molar ratio of 0.30, showcasing the system’s sensitivity in detecting PNG and underscoring the importance of selecting the appropriate LP/PC ratio to improve detection capabilities.

**Figure 8:**
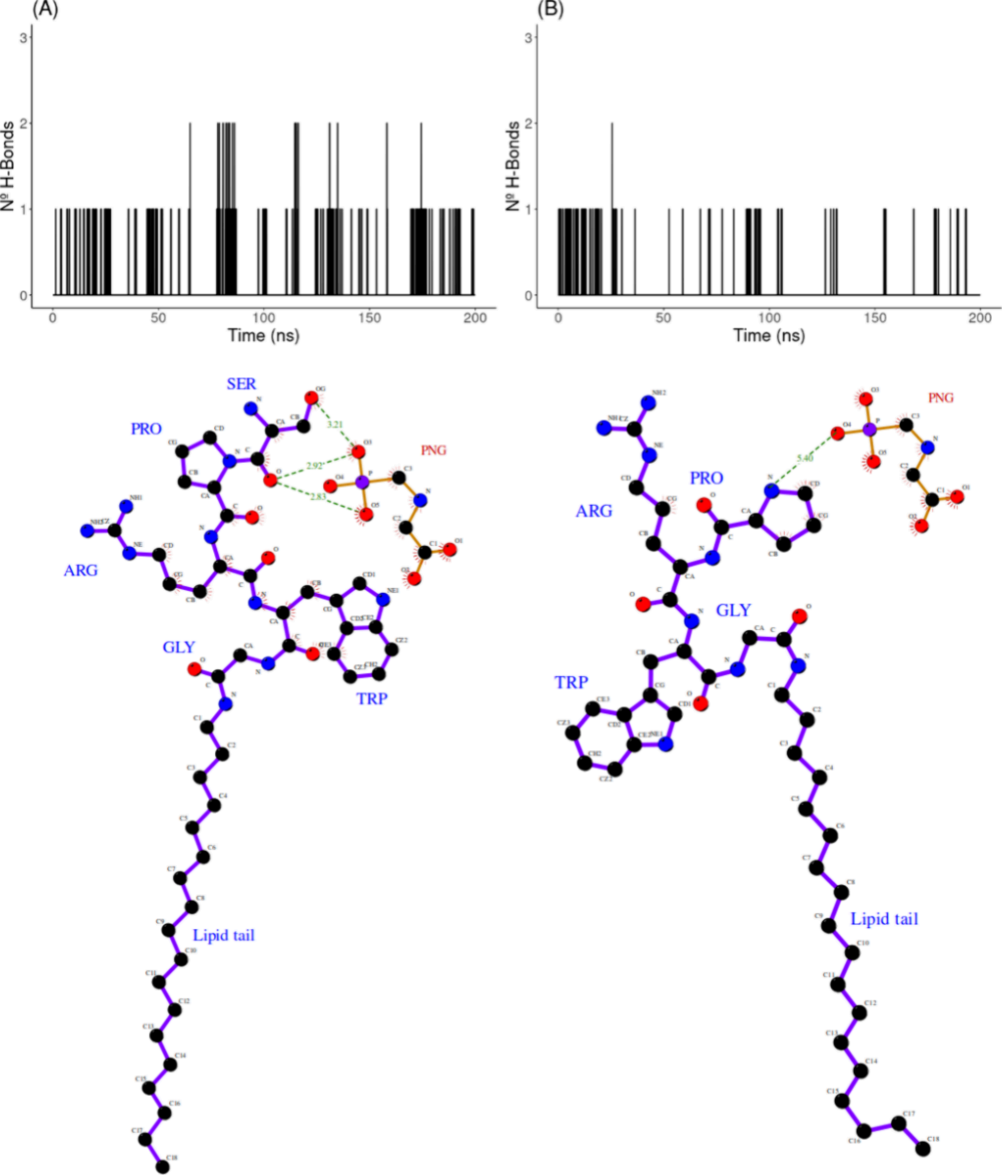
Time evolution (up) and 2D molecular rendering (down) of the number of H-bonds established between PNG and the terminal residue of LP along the MD trajectories of simulated systems at an [LP/PC] = 0.30 ratio. (A) **S**PRWG-(C_18_H_37_) and (B) **P**RWG-(C_18_H_37_). In 2D rendering, distances between the potential H-bond acceptor and donor atoms in PNG and LP are indicated for a representative time step in the simulation.

The achieved biosensor sensitivity is advantageous compared with a detection limit of approximately 0.3 μmol L^–1^, previously reported by us for a similar biosensor in aqueous media and with an [LP/PC] = 0.6 ratio.^27^ Even considering the dissimilar experimental conditions under which both sensors were tested, there is an order of magnitude difference between the two sensitivity measures. Notably, the lipid PC monolayer assayed in the prior investigation entailed an LP molecule lacking the terminal serine residue PRWG-(C_18_H_37_) instead of SPRWG-(C_18_H_37_) herein studied. A system encompassing the pesticide, the PRWG-(C_18_H_37_) compound, and the lipid monolayer at the usual [LP/PC] = 0.30 ratio was simulated to evaluate the effect of LP composition on its interaction with PNG. The nonbonded interaction energies and H-bond patterns between PNG and the monolayer with PRWG-(C_18_H_37_) were computed. The energy results (not shown) yielded a total nonbonded interaction energy between PNG and PRWG-(C_18_H_37_) almost 5 kcal/mol lower than the one verified when the serine residue is present in the structure (Table 2). H-bonds established exclusively between PNG and either the serine residue of SPRWG-(C_18_H_37_) or the proline residue of PRWG-(C_18_H_37_) species were measured (as shown in Figure 8). In this case, the number (and frequency) of hydrogen bonds of PNG with the terminal serine are doubled (at times tripled) compared with the terminal proline in the corresponding LP. Typically, H-bonds involve oxygen and nitrogen atoms of serine main and side chains and oxygen and nitrogen atoms of PNG (see Figure 8). These interactions are not seen between PNG and proline in PRWG-(C_18_H_37_), as the relevant polar centers are too far apart in space to form H-bonds. Thus, the terminal serine appears critical in stabilizing the interaction between PNG and the lipid monolayer. This could enable the observed superior sensitivity for PNG detection.

To evaluate the selectivity of a biosensor based on an LP/PC monolayer with a molar ratio of 0.30 for detecting PNG in water, experiments were conducted in the presence of potential interferents, such as carbaryl and malathion,^62,63^ as shown in Figure S11. Carbaryl and malathion are pesticides commonly found in enriched groundwater and plant extracts, which can interfere with the biosensor’s selectivity. Notably, malathion is classified as probably carcinogenic to humans.^64^ It was observed that adding these interferents reduces the charge transfer resistance compared to the scenario where only the LP/PC monolayer is present. Specifically, the charge transfer resistance for the carbaryl interferent matches that observed for the clean ITO surface. These findings confirm that the selected composition of the biosensor enables effective and selective detection of PNG. It is noteworthy that water samples containing various organic and inorganic compounds can complicate the analysis of pesticides. However, the demonstrated efficacy of the biosensor in discriminating against these interferents highlights its practical applicability in complex environmental settings. In our experiments with the lipopeptide-phosphatidylcholine monolayer, while we did not directly test in highly complex environmental matrices, the design of the lipopeptide to mimic acetylcholinesterase provides inherent specificity toward glyphosate due to structural and functional mimicry. This specificity is expected to minimize potential cross-reactivity with other substances typically found in environmental samples.

## Conclusions

This research demonstrated striking alterations in the structural and mechanical properties of the PC monolayer upon integrating the SPRWG-(C_18_H_37_) lipopeptide. These changes are marked by increased system flexibility and decreases in molecular area, surface pressure, and compressibility modulus, reflecting a significant modification of the monolayer physical properties. Surface potential measurements provided solid evidence of the pesticide’s direct interaction with the monolayer. Additionally, AFM topography images depicted the structural transformations caused by incorporating the lipopeptide into the monolayer and the subsequent introduction of PNG. The information gathered from in silico MD simulations proved very helpful in rationalizing, at a molecular level, the impact of LP on both the monolayer structure and the interaction with PNG. The electrochemical characterization following the application of the monolayer to ITO surfaces was a crucial component of our study. It enabled the detection of PNG at different concentrations, resulting in a calibration curve that exhibited a remarkable detection limit of 24 nmol L^–1^. This sensitivity enhancement was achieved by strategically including polar residues within the peptide sequence of the LP. Specifically, serine and arginine introduce functional groups capable of forming hydrogen bonds with the PNG. These interactions significantly boost the binding efficiency and specificity of the LP toward the pesticide. Consequently, this enhances the overall sensitivity of the detection system, making it an auspicious approach for the precise monitoring and quantification of glyphosate in environmental samples.

## Supporting Information Available

Illustrations of LP/PC monolayer biosensor construction steps on the ITO surface, time evolution of monolayer thickness, molecular models of lipid monolayers, compressibility modulus data for various molar ratios, surface pressure and potential measurements in the presence of PNG, radial distribution functions of PNG, AFM images of LB films on ITO, comprehensive electrochemical evaluations of modified ITO electrodes, and equations detailing electrochemical analysis and properties of interfaces under study (PDF)
