# Supplementary material for: Exploring the Use of a Lipopeptide in Dipalmitoylphosphatidylcholine Monolayers for Enhanced Detection of Glyphosate in Aqueous Environments
Source: Langmuir. 2024 Jun 22;40(26):13583–95. doi: 10.1021/acs.langmuir.4c01089 (PMC11223468; doi:10.1021/acs.langmuir.4c01089)
Supplement: Supplementary file 1 — la4c01089_si_001.pdf [file la4c01089_si_001.pdf]

Support Information

Exploring the Use of a Lipopeptide in Dipalmitoylphosphatidylcholine Monolayers for Enhanced Detection of Glyphosate in Aqueous Environments

Priscila S. Ferreira,[1#] Barbara B. Gerbelli,[1§#] Ana. C. H. Castro-Kochi,[1] Bruna Cortez,[1] Fabiola L. Castro,[1] Jorge Cantero,[2] Federico Iribarne,[2] Ian W. Hamley,[3] Wendel A. Alves[1,*]

[1]*Center for Natural and Human Sciences, Federal University of ABC, Santo André, 09210-580, Brazil.*

[2]*Bioinformatics and Computational Biology Group, Mathematics-DETEMA Department, Faculty of Chemistry, UdelaR, General Flores 2124, Montevideo 11600, Uruguay.*

[3]*Department of Chemistry, University of Reading, Reading RG6 6AD, UK.*

[§]*Present address: Diamond Light Source, Didcot, Oxfordshire, England, United Kingdom*

[#] *Co-contribution*

**Corresponding author:* wendel.alves@ufabc.edu.br

Figure S1. *Different views illustrate the steps in constructing the LP/PC monolayer biosensor. Panel (A) shows the procedure from a diagonal perspective, while panel (B) provides a frontal view. In both views, the ITO surface is used as a substrate, and the LP/PC monolayer is transferred onto it using the Langmuir-Schaefer technique. Subsequently, PNG is applied to the biosensor surface, followed by electrochemical analysis.* *3*

Figure S2. *Time evolution of monolayer thickness (in Å) along the MD trajectories of simulated systems. (A) LP/PC = 0, (B) [LP/PC] = 0.30.* *4*

Figure S3. *Molecular models (top and side views) of the simulated lipid monolayers. (A) [LP/PC]=0 and (B) [LP/LC] = 0.30. Carbon atoms of PC aliphatic tail are colored in grey, oxygen and nitrogen atoms from*

PC polar head in red and blue, respectively, and LP in green. The yellow square (in top views) represents the unit cell containing the actual atoms, surrounded by image atoms from the periodic cell extensions. 4
Figure S4. *Compressibility modulus for different LP to PC molar ratios LP/PC, ranging from 0.05 to 1.00.*.5
Figure S5. *Time evolution of APL (in Å²) along the MD trajectories of simulated systems. (A) [LP/PC]=0. (B) [LP/PC]=0.30.* 5
Figure S6. *Surface pressure isotherm as a function of $A^{-3/2}$ at different ratios for [LP/PC]=1.00 for different PNG concentrations.*............6
Figure S7. *The surface potential of LP/PC monolayers in the presence of 15 µmol L^{-1} PNG. The graph demonstrates an increase in the SP value upon adding LP to the monolayer, indicating potential interactions between the pesticide and the monolayer.* 6
Figure S8. *Radial distribution function (g(r)) of PNG along the MD trajectories of [LP/PC]=0 (red curve) and (B) [LP/LC]=0.30 (blue curve) systems. The distance was calculated concerning the polar group (head) of PC or the center of mass of serine residue in LP, respectively.*............7
Figure S9. *AFM images of LB films under various conditions: (A) ITO, (B) LP/PC on ITO.*............7
Figure S10. *Electrochemical evaluation of ITO electrode surface modification in the presence of LP/PC 0.30 of ratio molar (A) Cyclic voltammograms of the previously cleaned ITO electrode at different scan rates and the plot of peak current versus square root of the scan rate. Cyclic voltammograms of modified ITO electrodes of different mixtures and concentrations at different scan rates and the plot of peak current versus square root of scan rate, ranging from 10, 25, 50, 75, 100, 125, 150, 200, and 250 mV s^{-1}. (B) LP/PC+PNG (1 µmol L^{-1}); (C) LP/PC+PNG (3 µmol L^{-1}); (D) LP/PC+PNG (5 µmol L^{-1}); (E) LP/PC+PNG (8 µmol L^{-1}) and (F) LP/PC+PNG (10 µmol L^{-1}). Electrolyte: solution of 5 mmol L^{-1} $K_4Fe(CN)_6/K_3Fe(CN)_6$ in 0.1 mol L^{-1} KCl.*............9
Figure S11. *Electrochemical analysis illustrating the modification of ITO with a 0.30 molar ratio LP/PC monolayer in the presence of interfering pesticides. The Nyquist plot shows the response of unmodified ITO (black line), ITO modified with the monolayer (red line), ITO modified with the monolayer in the presence of Carbaryl (pink line), and ITO modified with the monolayer in the presence of the interferent Malathion (green Line).*............10

Equation S1. Where: Ip is the peak current, n is the number of electrons transferred during oxidation or reduction, A is the electroactive area of the electrode (cm^2), D is the diffusion coefficient ($cm^2 \cdot s^{-1}$), C is the concentration of the electroactive species ($mol \cdot cm^{-3}$), and V is the scan rate ($V \cdot s^{-1}$). Through this equation, we can determine the electroactive area of the electrode using a solution of 5 mmol L^{-1} $K_4Fe(CN)_6/K_3Fe(CN)_6$ in 0.1 mol L^{-1} KCl as a probe............*9*
Equation S2. For this equation, the first term refers to the angular coefficient of the lines obtained in the graph of peak current versus the square root of the scan rate. Where: $n = 1$, $C = 5 \times 10^{-6}$ $mol \cdot cm^{-3}$, D is the diffusion coefficient of potassium ferricyanide, equal to 6.39×10^{-6} $cm^2 \cdot s^{-1}$. *10*

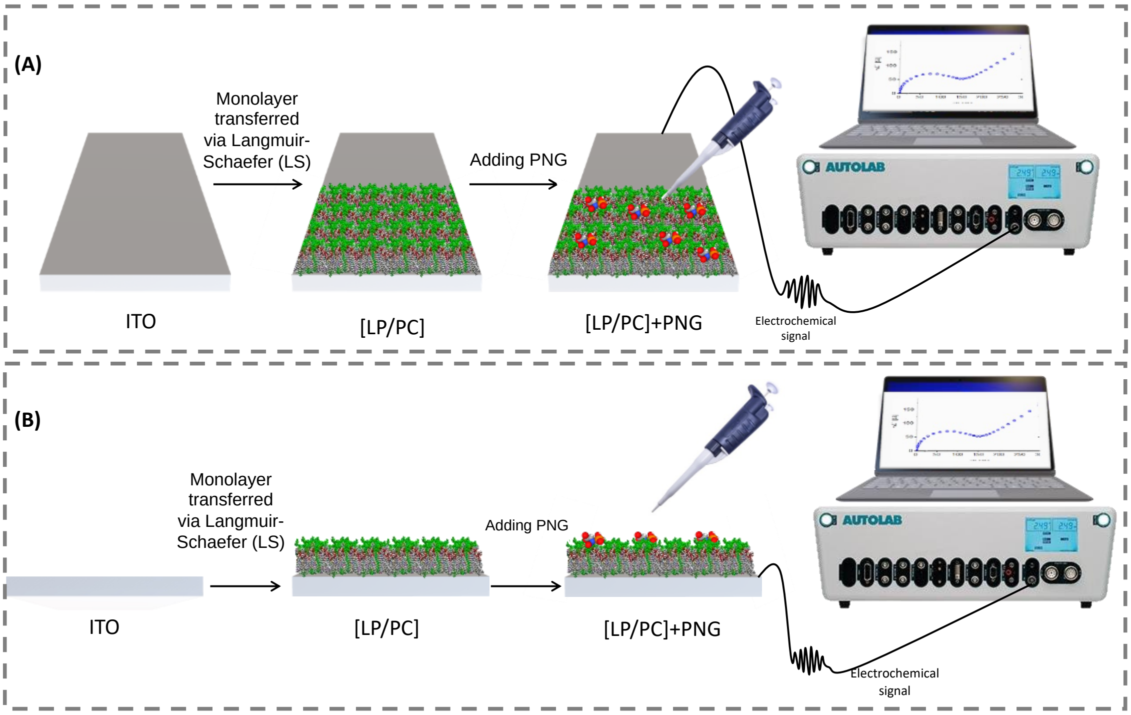

Figure S1. *Different views illustrate the steps in constructing the LP/PC monolayer biosensor. Panel (A) shows the procedure from a diagonal perspective, while panel (B) provides a frontal view. In both views, the ITO surface is used as a substrate, and the LP/PC monolayer is transferred onto it using the Langmuir-Schaefer technique. Subsequently, PNG is applied to the biosensor surface, followed by electrochemical analysis.*

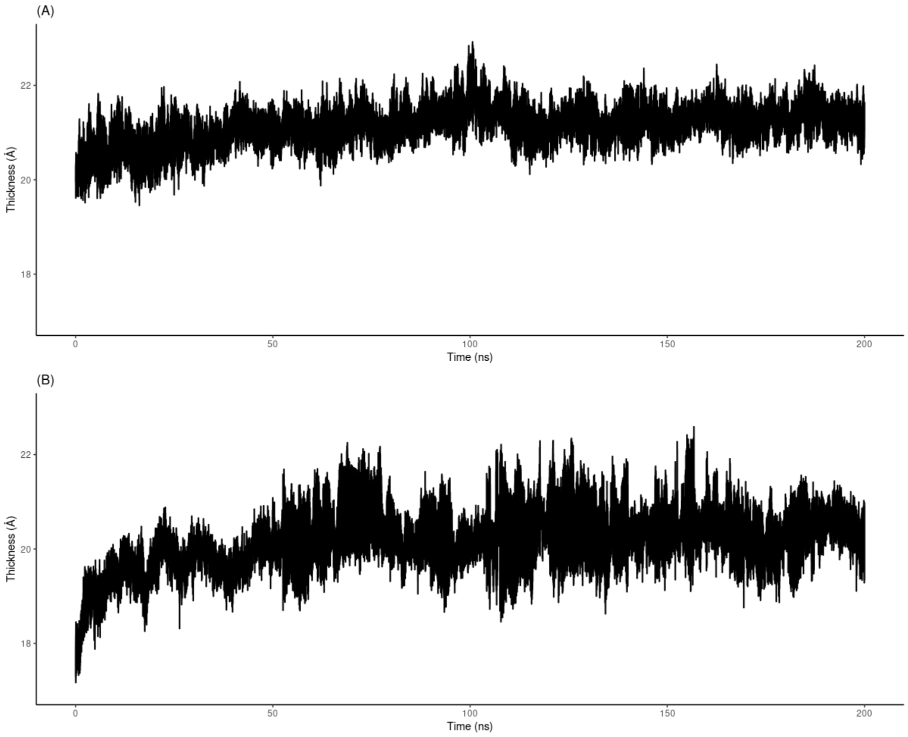

Figure S2. *Time evolution of monolayer thickness (in Å) along the MD trajectories of simulated systems. (A) [LP/PC]=0, (B) [LP/PC] = 0.30.*

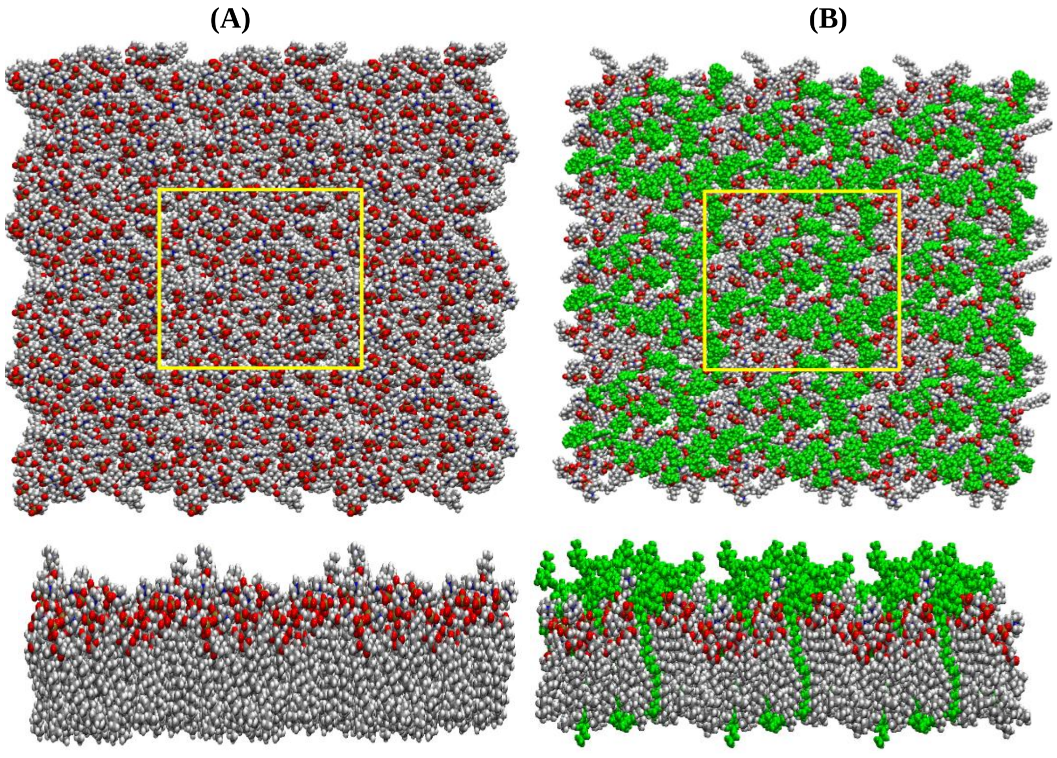

Figure S3. *Molecular models (top and side views) of the simulated lipid monolayers. (A) [LP/PC]=0 and (B) [LP/LC] = 0.30. Carbon atoms of PC aliphatic tail are colored in grey, oxygen and nitrogen atoms from PC polar head in red and blue, respectively, and LP in green. The yellow square (in top views) represents the unit cell containing the actual atoms, surrounded by image atoms from the periodic cell extensions.*

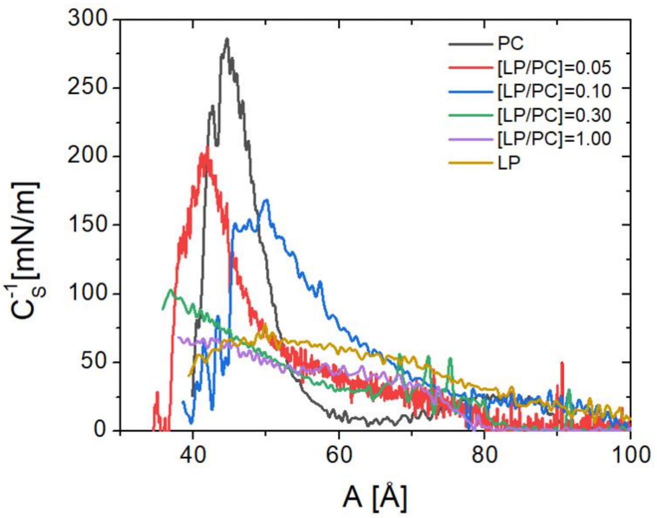

Figure S4. *Compressibility modulus for different LP to PC molar ratios LP/PC, ranging from 0.05 to 1.00.*

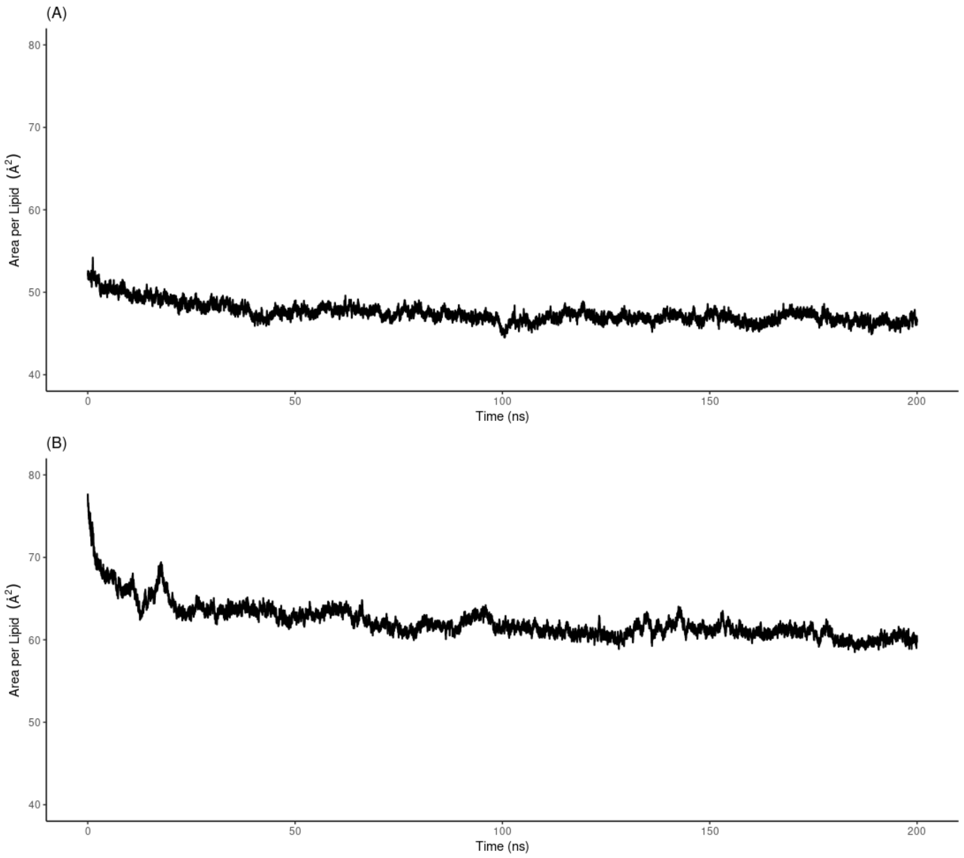

Figure S5. *Time evolution of APL (in Å^2) along the MD trajectories of simulated systems. (A) [LP/PC]=0. (B) [LP/PC]=0.30.*

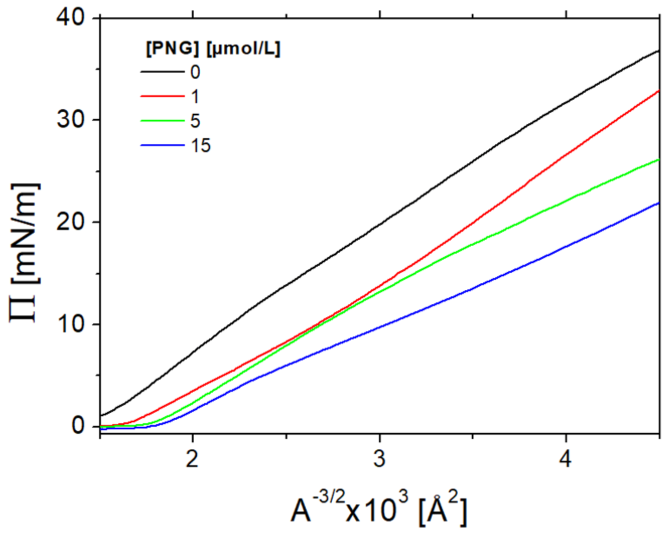

Figure S6. *Surface pressure isotherm as a function of* $A^{-3/2}$ *at different ratios for [LP/PC]=1.00 for different PNG concentrations.*

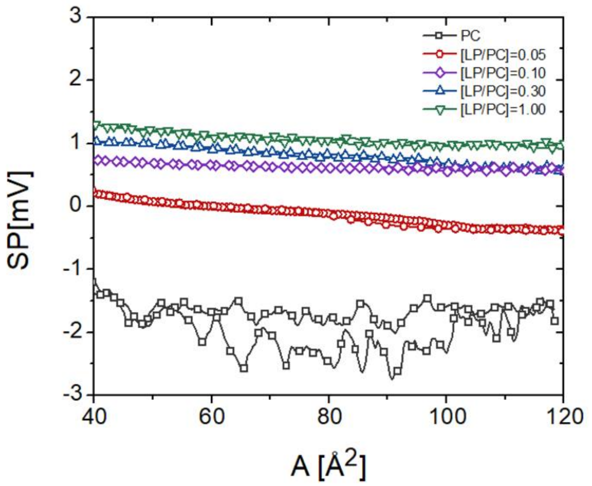

Figure S7. *The surface potential of LP/PC monolayers in the presence of 15 μmol* L^{-1} *PNG. The graph demonstrates an increase in the SP value upon adding LP to the monolayer, indicating potential interactions between the pesticide and the monolayer.*

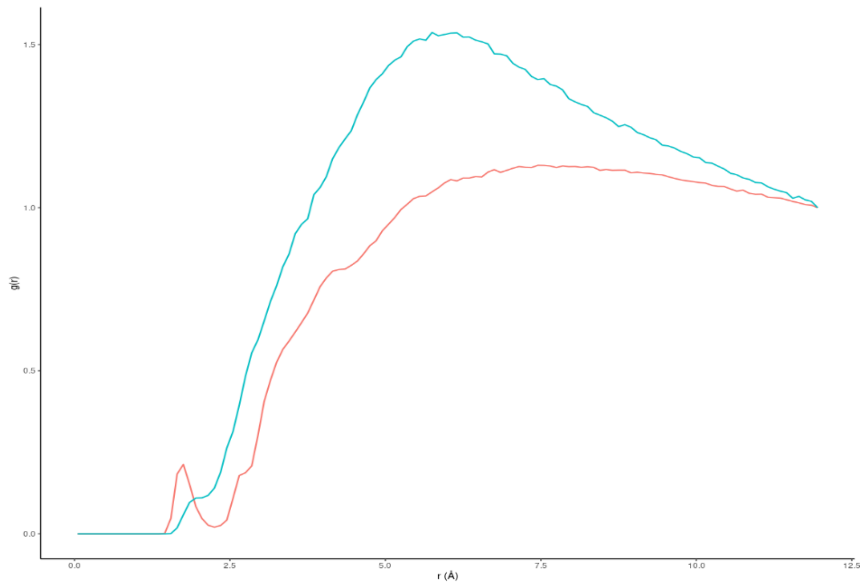

Figure S8. *Radial distribution function (g(r)) of PNG along the MD trajectories of [LP/PC]=0 (red curve) and (B) [LP/LC]=0.30 (blue curve) systems. The distance was calculated concerning the polar group (head) of PC or the center of mass of serine residue in LP, respectively.*

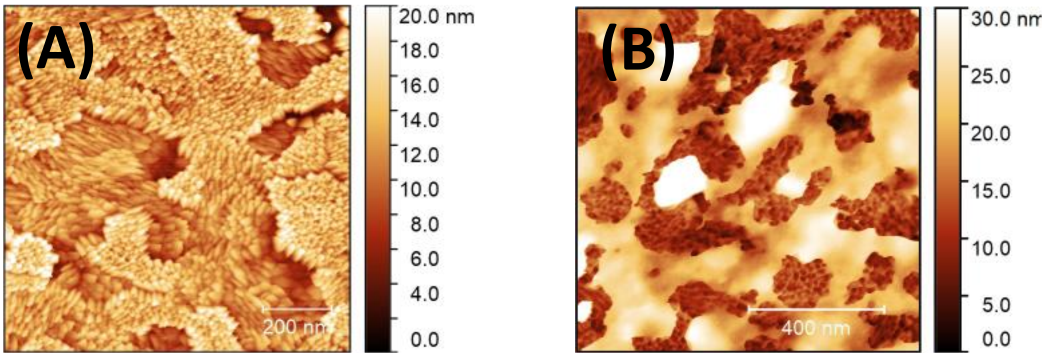

Figure S9. *AFM images of LB films under various conditions: (A) ITO, (B) LP/PC on ITO.*

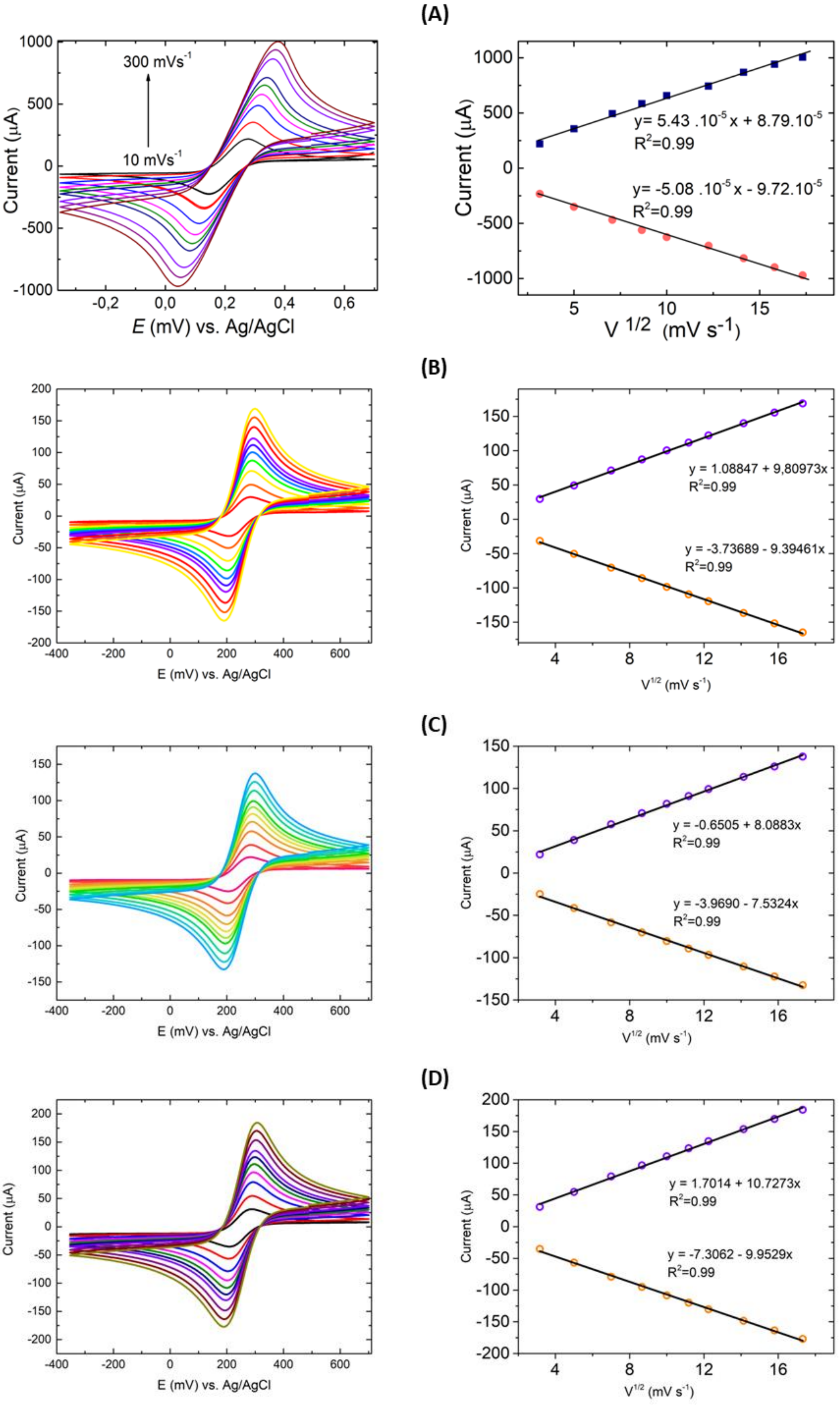
(A)
300 mVs-1
10 mVs-1
Current (μA)
E (mV) vs. Ag/AgCl
y= 5.43 .10-5 x + 8.79.10-5
R2=0.99
y= -5.08 .10-5 x - 9.72.10-5
R2=0.99
Current (μA)
V 1/2 (mV s-1)
(B)
Current (μA)
E (mV) vs. Ag/AgCl
y = 1.08847 + 9,80973x
R2=0.99
y = -3.73689 - 9.39461x
R2=0.99
Current (μA)
V1/2 (mV s-1)
(C)
Current (μA)
E (mV) vs. Ag/AgCl
y = -0.6505 + 8.0883x
R2=0.99
y = -3.9690 - 7.5324x
R2=0.99
Current (μA)
V1/2 (mV s-1)
(D)
Current (μA)
E (mV) vs. Ag/AgCl
y = 1.7014 + 10.7273x
R2=0.99
y = -7.3062 - 9.9529x
R2=0.99
Current (μA)
V1/2 (mV s-1)

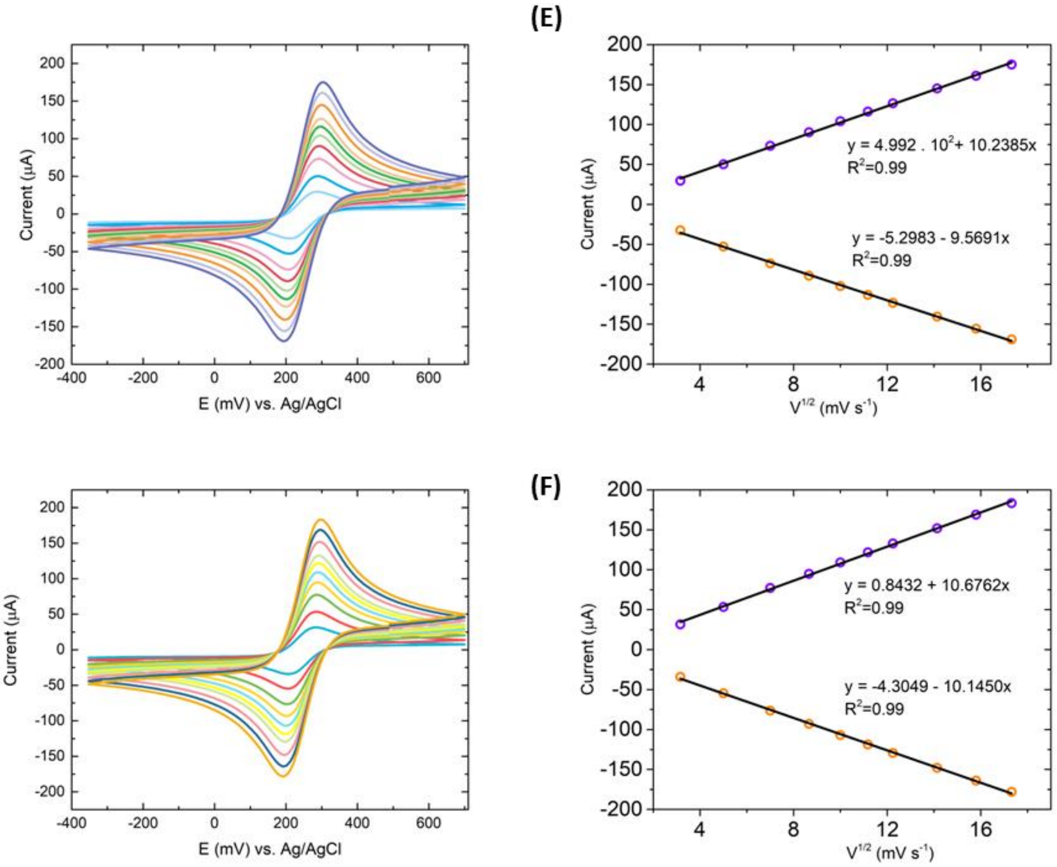

Figure S10. *Electrochemical evaluation of ITO electrode surface modification in the presence of LP/PC 0.30 of ratio molar (A) Cyclic voltammograms of the previously cleaned ITO electrode at different scan rates and the plot of peak current versus square root of the scan rate. Cyclic voltammograms of modified ITO electrodes of different mixtures and concentrations at different scan rates and the plot of peak current versus square root of scan rate, ranging from 10, 25, 50, 75, 100, 125, 150, 200, and 250 mV s^{-1}. (B) LP/PC+PNG (1 μmol L^{-1}); (C) LP/PC+PNG (3 μmol L^{-1}); (D) LP/PC+PNG (5 μmol L^{-1}); (E) LP/PC+PNG (8 μmol L^{-1}) and (F) LP/PC+PNG (10 μmol L^{-1}). Electrolyte: solution of 5 mmol L^{-1} $K_4Fe(CN)_6/K_3Fe(CN)_6$ in 0.1 mol L^{-1} KCl.*

Equation S1:

$$I_p = (2.69 \times 10^5) n^{3/2} A D^{1/2} C V^{1/2}$$

Here: I_p is the peak current, n is the number of electrons transferred during oxidation or reduction, A is the electroactive area of the electrode (cm^2), D is the diffusion coefficient ($cm^2 \cdot s^{-1}$), C is the concentration of the electroactive species ($mol \cdot cm^{-3}$) and V is the scan rate ($V \cdot s^{-1}$). Through this equation, we can determine the electroactive area of the electrode using a solution of 5 mmol L^{-1} $K_4Fe(CN)_6/K_3Fe(CN)_6$ in 0.1 mol L^{-1} KCl as a probe.

Equation S2:

$$A = \frac{I_p}{V^{1/2}} \times \frac{1}{2.69 \times 10^5 n^{3/2} D^{1/2} C}$$

For this equation, the first term refers to the angular coefficient of the lines obtained in the graph of peak current *versus* the square root of the scan rate. Where: $n = 1$, $C = 5 \times 10^{-6}\ mol{\cdot}cm^{-3}$, D is the diffusion coefficient of potassium ferricyanide, equal to $6.39 \times 10^{-6}\ cm^2{\cdot}s^{-1}$.

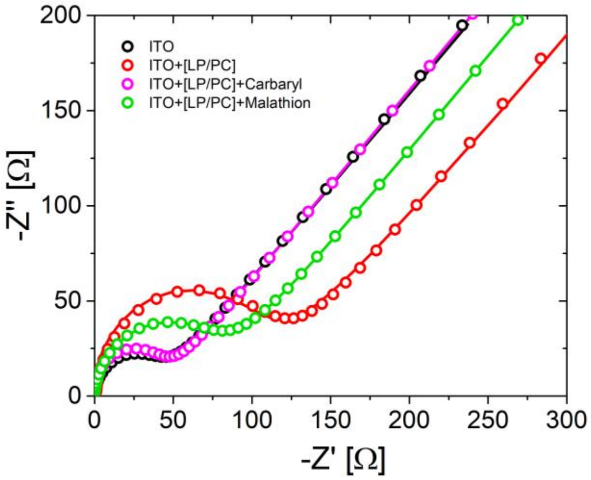

Figure S11. *Electrochemical analysis illustrating the modification of ITO with a 0.30 molar ratio LP/PC monolayer in the presence of interfering pesticides. The Nyquist plot shows the response of unmodified ITO (black line), ITO modified with the monolayer (red line), ITO modified with the monolayer in the presence of Carbaryl (pink line), and ITO modified with the monolayer in the presence of the interferent Malathion (green Line).*
